# Oscillatory fluid flow enhanced mineralization of human dental pulp cells

**DOI:** 10.3389/fbioe.2025.1500730

**Published:** 2025-01-15

**Authors:** Witsanu Yortchan, Sasima Puwanun

**Affiliations:** Department of Preventive Dentistry, Division of Pediatric Dentistry, Faculty of Dentistry, Naresuan University, Phitsanulok, Thailand

**Keywords:** bone, tissue engineering, orofacial defect, dental pulp, fluid flow, mesenchymal stem cells

## Abstract

The purpose of this study is to evaluate the optimum frequency of oscillatory fluid flow (OFF) for increasing osteogenesis in human dental pulp cells (DPCs) in an incubating rocking shaker. DPCs from 3 donors were cultured in an osteogenic induction medium (OIM) and mechanical stimulation was applied using an incubating rocking shaker at frequencies of 0 (control), 10, 20, 30, and 40 round per minute (RPM) for 1 h/day, 5 days/week. Cell proliferation was measured using total protein quantification, and osteogenic activity was measured by alkaline phosphatase (ALP) activity, calcium deposition, and collagen production on days 7, 14, and 21 of culture. Results of DPCs morphology in the 30 RPM group were more clustered and formed interconnections between cells. Results of DPC proliferation and collagen production showed no significant differences between the experiment groups. The ALP activity on day 7 and 14, and calcium deposition on day 21 of the 30 RPM group were significantly higher than the control groups. Thus 30 RPM is likely an effective frequency for increasing calcium deposition. This study uses strategies in Tissue Engineering followed the research topic about an application of human cells to stimulate oral and maxillofacial hard tissue regeneration. In the future, the mineralization of DPCs could be enhanced by using an incubating rocking shaker at 30 RPM in the lab to create a cell sheet. The mineralized cell sheet could then be implanted into the patient for bone repair of orofacial defects.

## 1 Introduction

Tissue engineering has shown much promise in the repair of craniofacial defects. This strategy would solve problems associated with currently used bone grafting such as donor site morbidity, immune rejection, and pathogen cross-contamination. A previous clinical study compared the quantity of bone formation at the maxillary sinus after implantation of either hydroxyapatite (HA) granules alone or a mixture of HA granules and cortical bone graft from the chin. The results showed more new bone formation in the mixture group than in the group with HA granules alone, approximately 44.4% and 20.3%, respectively (Moy et al., 1993). This showed that the scaffold that included cells could increase a bone repair success rate in a bone tissue engineering procedure, compared to the scaffold control alone.

Dental pulp cells (DPCs) from the dental pulp tissue have been used as an alternative cell source for autologous mesenchymal stem cells (MSCs) to repair craniofacial bone defects. Many studies have found that dental pulp tissue contains dental pulp derived mesenchymal stem cells (DPSCs) (Chen et al., 2022; Masuda et al., 2021; Zhao et al., 2020). These cells are responsible for producing dentine and nerve fibers (Iezzi et al., 2019). Dental pulp tissue can be easily harvested from extracted teeth, such as removed wisdom teeth or non-functional teeth, and then explanted to produce DPSCs *in vitro*. Also DPSCs can be stored in liquid nitrogen for a long time while maintaining the MSC characteristics. After storage in liquid nitrogen, MSCs still undergo normal osteogenic, adipogenic, or chondrogenic differentiation (Sonmez Kaplan et al., 2023). DPCs are able to express important bone markers, including osteonectin, bone sialoprotein, osteocalcin, fibronectin, and alkaline phosphatase (ALP) (Gronthos et al., 2000). DPCs can also form new lamellar bone with vascularization and osteocytes contained in haversian canals, *in vivo* (Paino et al., 2017). Thus DPCs can be used as a cell source for craniofacial repair in the dental and periodontal fields (Jeon and Kim, 2014).

Dentin is a dynamic tissue which is able to adjust its own architecture. The dentinal fluid contained in the dentinal tubules is affected by mechanical stimuli. Reparative dentin is formed as a reaction to tooth wear (from chewing), which causes the movement of dentinal fluid. The term “mechanotransduction” refers to a cell process in which mechanical stimulation causes a chemical response in cells. Since fluid plays an important role as a constituent of dentin, fluid flow is also an influential mechanical stimulus for cells in the dental pulp. The cytoplasm of odontoblasts extends to establish communication between dentin and the dental pulp (Khurshid et al., 2021).

Stavenschi et al. (2017) demonstrated the use of mechanical stimulation, specifically fluid flow, for enhancing osteogenic differentiation, *in vivo*. Oscillatory fluid flow (OFF) generated by a standard rocking shaker has been used to increase osteogenic differentiation of MSCs (Delaine-Smith et al., 2012; Zhou et al., 2010). Fluid flow-induced shear stress similarly occurs to osteocytes naturally in the canalicular system (Wittkowske et al., 2016). Fluid flow has also been shown to increase calcium content and matrix distribution in a mouse osteoblastic precursor cell line, MC3T3-E1 (Killinger et al., 2023). Many studies have investigated and reported on a wide range of fluid flow between 0.1 and 2 Pa (Nauman et al., 2001; Stavenschi et al., 2017). The exact fluid flow required to enhance mineralization in DPCs is difficult to determine. An improved understanding of mechanical stimulation-induced mineralization is needed. The optimal frequency for mechanical stimulation of DPCs to increase mineralization using OFF generated by an incubating rocking shaker is still unknown.

The purpose of this study is to evaluate the optimum frequency to create shear stress for increasing osteogenesis in human dental pulp cells in an incubating rocking shaker.

## 2 Methods and materials

All culture consumables and chemicals were obtained from Sigma-Aldrich (Burlington, Massachusetts, United States) and used as supplied unless otherwise stated.

### 2.1 Cell isolation

DPCs were isolated from the dental pulp tissue of non-functional teeth collected with written informed consent from patients undergoing maxillofacial surgery at the Dental Hospital of Naresuan University in Phitsanulok, Thailand. This study was conducted with ethical approval from the Ethics Committee of Naresuan University (reference number IRB 0801/62, October, 2019). The recruitment period for this study started 14th November 2019 and ended 12th September 2020. The dental pulp tissues were isolated from the teeth within 24 h after extraction. This study involves three donors, and the following cell isolation procedure was carried out three separate times, one time for each donor. Each time, the tissues were removed and rinsed with phosphate-buffered saline (PBS) containing 100 mg/mL penicillin-streptomycin (Caisson labs, Smithfield, Montana, United States). The tissues were then cut into small pieces, and 4 mg/mL dispase solution (Gibco, Billings, Montana, United States) was added. After that, the cells in solution were centrifuged and the supernatant was removed. 5 mL of fresh basal culture media (BCM) was added to the centrifuge tube. The BCM consisted of alpha-MEM (Hyclone, Cytiva Life Sciences, Marlborough, Massachusetts, United States) supplemented with 10% fetal bovine serum (v/v) (Capricorn, Ebsdorfergrund, Germany), 2 mM L-glutamine (Hyclone), 100 mg/mL penicillin-streptomycin according to the method of Vishwanath et al. (2013). The contents of the centrifuge tube were transferred to a 25 cm^2^ tissue-culture flask (Thermo Fisher Scientific, Waltham, Massachusetts, United States) and then cultured in an incubator (Thermo Fisher Scientific) for 7 days at 37°C in 5% CO_2_ in a humidified atmosphere. Fresh BCM was added to the flasks every 2–3 days during the 7-day incubation period. After incubation, the culture media and non-adherent cells were removed. The adherent cells are hereafter referred to as “dental pulp cells (DPCs).” A total minimum of 10^5^ DPCs were divided approximately between 10 new flasks with fresh BCM and cultured for another 7 days under the previous conditions in order to multiply the cells. That was passage 1. Finally, the cells were then ready for seeding onto samples. The cell isolation procedures for the three donors were carried out in succession, but all three were completed before beginning the next step.

### 2.2 Expression assessment of DPCs surface markers

The expression levels of surface markers on DPCs, specifically CD90, CD105, CD146, and CD45, were detected using antibodies from the human MSC multi-color flow cytometry kit (R&D Systems, Abingdon, United Kingdom), which includes anti-CD90 Allophycocyanin (APC), anti-CD105 Peridinin Chlorophyll Protein Complex (Per-CP), anti-CD146 Carboxyfluorescein (CFS), and anti-CD45 Phycoerythrin (PE). Additionally, anti-human CD105 Brilliant Violet 421™ (BV) (BioLegend, San Diego, California, United States) was used for CD105. The stained cells were resuspended in flow cytometry solution (containing 0.1% bovine serum albumin, 0.1% sodium azide in PBS) in an appropriate volume. The cells were evaluated using an LSR II flow cytometer (BD Biosciences, Oxford, United Kingdom).

### 2.3 Dental pulp cell culture in osteogenic induction media

DPCs from the 3 donors were seeded separately into 6-well plates (Corning, Steuben County, New York, United States) at a density of 10^4^ cells per well along with 2 mL of BCM and incubated under the previously described conditions for 24 h in order to allow the cells to attach to the well plate. After 24 h, the BCM was replaced with OIM which consisted of BCM supplemented with 50 μg/mL of ascorbic acid-2-phosphate, 5 mM of beta-glycerophosphate, and 10 nM of dexamethasone. The cells in OIM were then incubated again for 21 days. The cells were observed daily for cell morphology, color of the medium, and cell density. The OIM was replaced every 2–3 days. That was passage 2. De Bari et al. (2006) showed that DPCs between passages 2–5 are viable for efficient osteogenic differentiation.

### 2.4 Mechanical force stimulation

**FIGURE 1:**
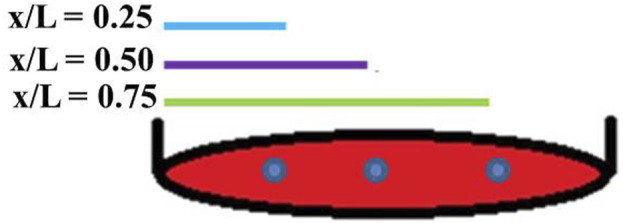
The three different points (blue dots) on the circular culture surface of the 6-well plate for one cycle were calculated for OFF stress in parallel with the diameter at ***x/L*** = 0.25, 0.5, and 0.75. Here, ***x*** represents the distance from the reference point to the edge of the well, and ***L*** is the diameter of the well along the axis of rotation.

OFF is employed in this study for long term cell culture. Media fluid stimulates the cells in 6-well plates placed in an incubating rocking shaker (VWR International, Radnor, Pennsylvania, United States). OFF applies shear stress to the bottom of the well and this stress was calculated at three separate points (Figure 1) using the lubrication-base model equation (Equation 1) previously reported by Zhou et al. (2010).

$$|{\tilde{\tau }}_{w}\left|=\right.\frac{\pi \mu \theta_{\max}}{2\delta^{2}T}$$ (1)

Where ***µ*** is the fluid viscosity (10^–3^ Pa s), ***θ***_***max***_ max is the maximum flip angle (6.8° in the 2 mL of media condition), ***δ*** is the ratio between the depth of fluid and the length of the well, and ***T*** is the period of time per cycle.

On day 4 of cell culture passage 2, the well plates began undergoing mechanical stimulation in the incubating rocking shaker. There were five experimental groups differing, by the RPM applied: 0 (a static group), 10, 20, 30, and 40 RPM. Mechanical stimulation occurred for 1 h a day, 5 days per week, at 37°C, as per the schedule used by Delaine-Smith et al. (2012).

In this study, the shear stress values generated at 10, 20, 30, and 40 RPM were 0.50, 0.92, 1.24, and 1.34 Pa, respectively.

### 2.5 Total protein quantification

Total protein quantification was carried out using the Micro BCA™ protein assay kit (Thermo Fisher Scientific) (Johnson, 2012). The samples were added to the cell digestion buffer to extract protein, stored at −80°C for 10 min, incubated at 37°C for 15 min, freeze-thawed three times, and vortexed for 15 s. The samples were combined with bicinchoninic acid (BCA) working reagent, and 200 µL of this BCA working reagent mixture was transferred into a 96-well plate and incubated at 37°C for 30 min. The samples were measured at the wavelength of 562 nm using a spectrophotometer (Bio-Rad, Hercules, California, United States). Total protein quantification was used for data normalization. All fold changes in the total protein quantification are relative to the result of the 0 RPM group on day 7 (the control).

### 2.6 Alkaline phosphatase activity measurement

ALP is a cell enzyme that can be used to evaluate matrix maturation during osteogenic differentiation. ALP hydrolyzes pyrophosphate which is present in the OIM and this produces inorganic phosphate to enhance mineralization. Samples were washed twice with PBS. 500 μL of cell lysate buffer was added to the cells and left for 20 min at 37°C before scraping to remove the cell lysate. The cell lysates were freeze-thawed three times, vortexed, and centrifuged. The cell lysates were mixed with Alkaline Phosphatase Yellow Liquid Substrate based on p-nitrophenol phosphate. The spectrophotometer was used to measure the absorbance at 405 nm every minute for 30 min. The enzyme activity was calculated as nano-mol of para-nitrophenol per min (nmol pNP/min). The ALP activity was normalized by divided by the protein quantification from the same sample. All fold changes in ALP activity are relative to the result of the 0 RPM group on day 7.

### 2.7 Calcium mineralization staining

Calcium mineralization was assessed using the alizarin red staining method. This method is commonly used to evaluate the effect of OFF on the mineralization of human MSCs (Lim et al., 2021; Mishra et al., 2023). Calcium mineralization was measured on day 21 of the experiment. First the cells were washed twice with PBS and once more with distilled water (dH_2_O). They were fixed with 10% formalin for 20 min and then rinsed with dH_2_O two more times. A staining solution of 1 mg/mL alizarin red in dH_2_O was prepared and adjusted to pH 4.1 with ammonium hydroxide. The cells were then stained for 20 min at room temperature. The stained samples were dissolved by adding 500 µL 5% perchloric acid in double distilled water and left in the unheated but moving rocking shaker for 30 min. 150 μL of the eluted solutions were measured at 570 nm in the spectrophotometer (Kaewkhaw et al., 2011). All fold changes in calcium deposition are relative to the result of the 0 RPM group.

### 2.8 Collagen staining

Collagen production was measured on days 14 and 21 of the experiment. The media was removed and the cells were washed three times with PBS. The samples were fixed with 10% formalin for 20 min and then washed again with PBS. Next the cells were stained with 0.1% picrosirius red solution for 18 h in the unheated but moving rocking shaker. The dye solution was removed, and the cells were air-dried and then destained with 0.2 M sodium hydroxide and methanol in a ratio 1:1 for 15 min in the rocking shaker. 200 μL of each sample’s eluted solution was measured at 490 nm in the spectrophotometer. All fold changes in collagen production are relative to the result of the 0 RPM group.

### 2.9 Statistics

Data are expressed as mean values ± standard error of the mean (SEM). The number of replicates is stated in the figure legend. “N” represents a biological repeat (separate experiment), and “n” represents a technical repeat (duplicate samples within one experiment). Statistical analysis was performed using SPSS (IBM SPSS statistics 23). Total protein quantification, ALP activity, total calcium, and collagen production were analyzed using independent samples in the Kruskal–Wallis test. The differences were statistically significant when the p-value was less than or equal to 0.05 *(p ≤ 0.05)*.

## 3 Results

### 3.1 DPCs surface marker expression

**FIGURE 2:**
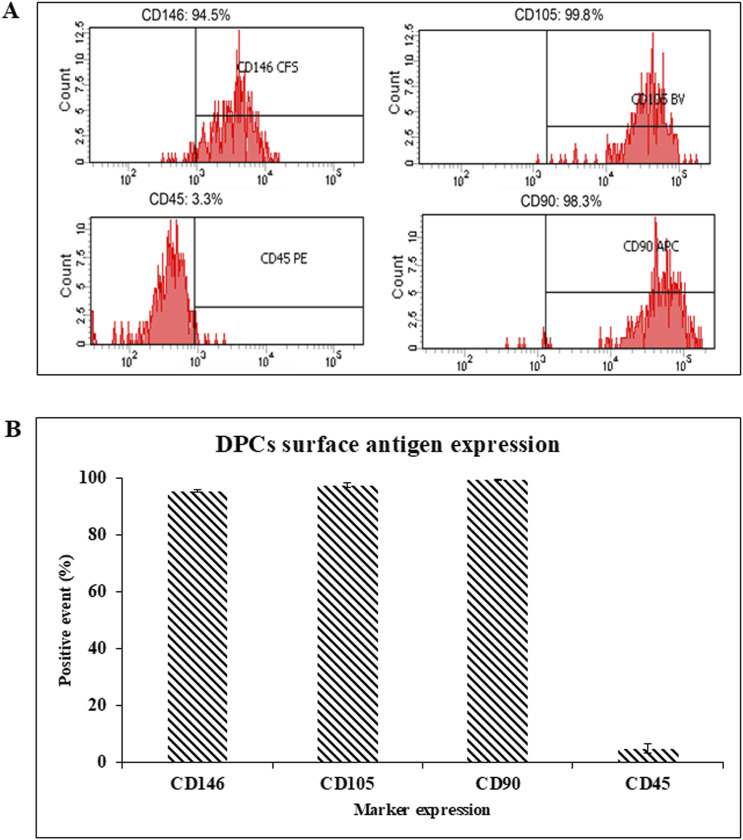
**(A)** The representative surface antigen expression patterns of DPCs were measured using flow-assisted cell sorting. **(B)** Summary of the surface antigen expression patterns of the DPCs from 3 donors.

Flow cytometry was used to characterize cell properties and identify biomarkers. The average results from 3 donors showed that the DPCs express the tested MSC markers at about 99.43, 97.43, and 95.40% for CD90, CD105, CD146, respectively. Meanwhile the average expression of the hematopoietic marker (CD45) from the 3 donors was 4.8% in the DPCs (Figure 2). This implies that DPCs meet the minimal requirement of MSCs specified by the International Society for Cellular Therapy in 2006 (Dominici et al., 2006).

### 3.2 Osteogenic differentiation of DPCs

**FIGURE 3:**
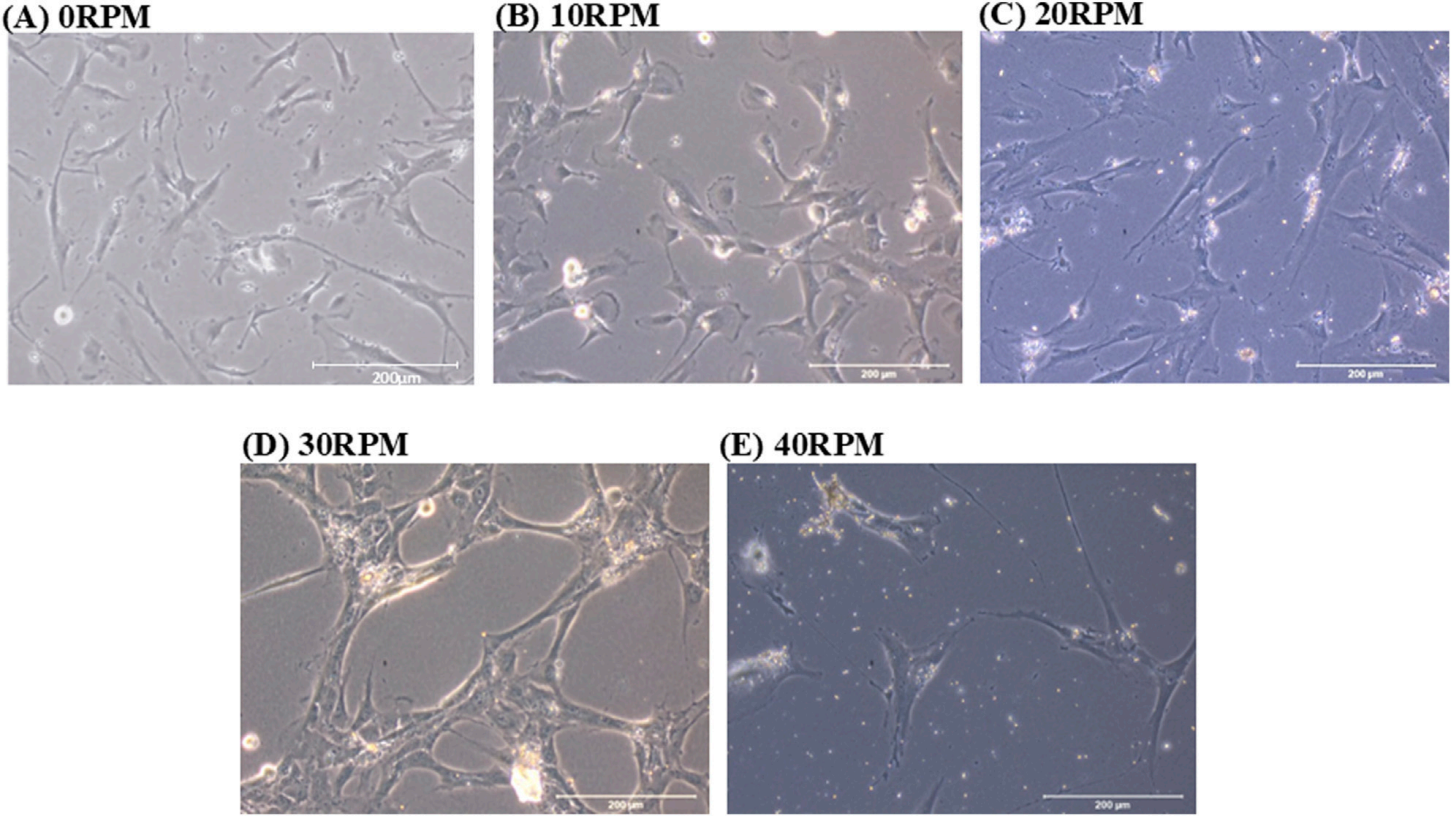
Phase contrast images show the cell morphology of human DPCs in OIM after 7 days. **(A)** In the 0 RPM (control) group, the cells were fibroblast-like and scattered individually. **(B)** In the 10 RPM group and **(C)** in the 20 RPM group, cells were scattered individually, similar to the control group. **(D)** In the 30 RPM group, the cells were more clustered and gathered together. **(E)** In the 40 RPM group, cells were polygonal in shape and more scattered in large spaces compared to the other groups. The scale bar represents 200 μm.

DPCs morphologies were observed at 0, 10, 20, 30 and 40 RPM in OIM for 3, 7, and 14 days of culture. On day 3 of culture, the cells in all groups were fibroblast-like and scattered individually. On day 7 of culture, in the 0 RPM (control) group, the cells were fibroblast-like and scattered individually. In the 10 RPM and 20 RPM groups, cells were scattered individually, similar to the control group. In the 30 RPM group, the cells were more clustered and gathered together. In the 40 RPM group, cells were polygonal in shape and more scattered in large spaces compared to the other groups. On 14 days of culture, the 20 RPM, 30 RPM, and 40 RPM groups reached confluence (Figure 3).

**FIGURE 4:**
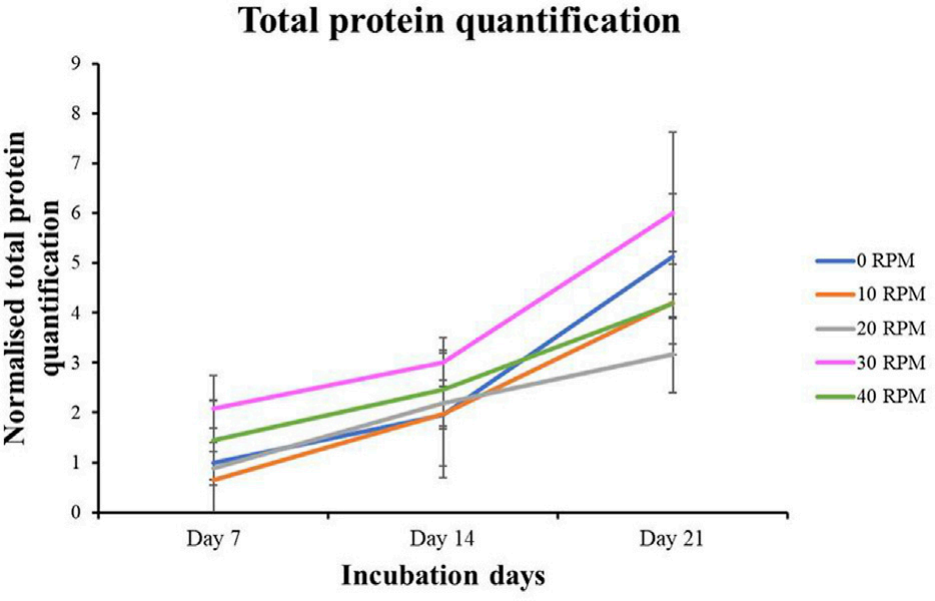
Total protein quantification of DPCs in the osteogenic induction medium was measured by BCA protein assay for different OFF frequencies on 7, 14, and 21 days of culture. Data are presented as mean ± SEM (N = 3, n = 3).

**FIGURE 5:**
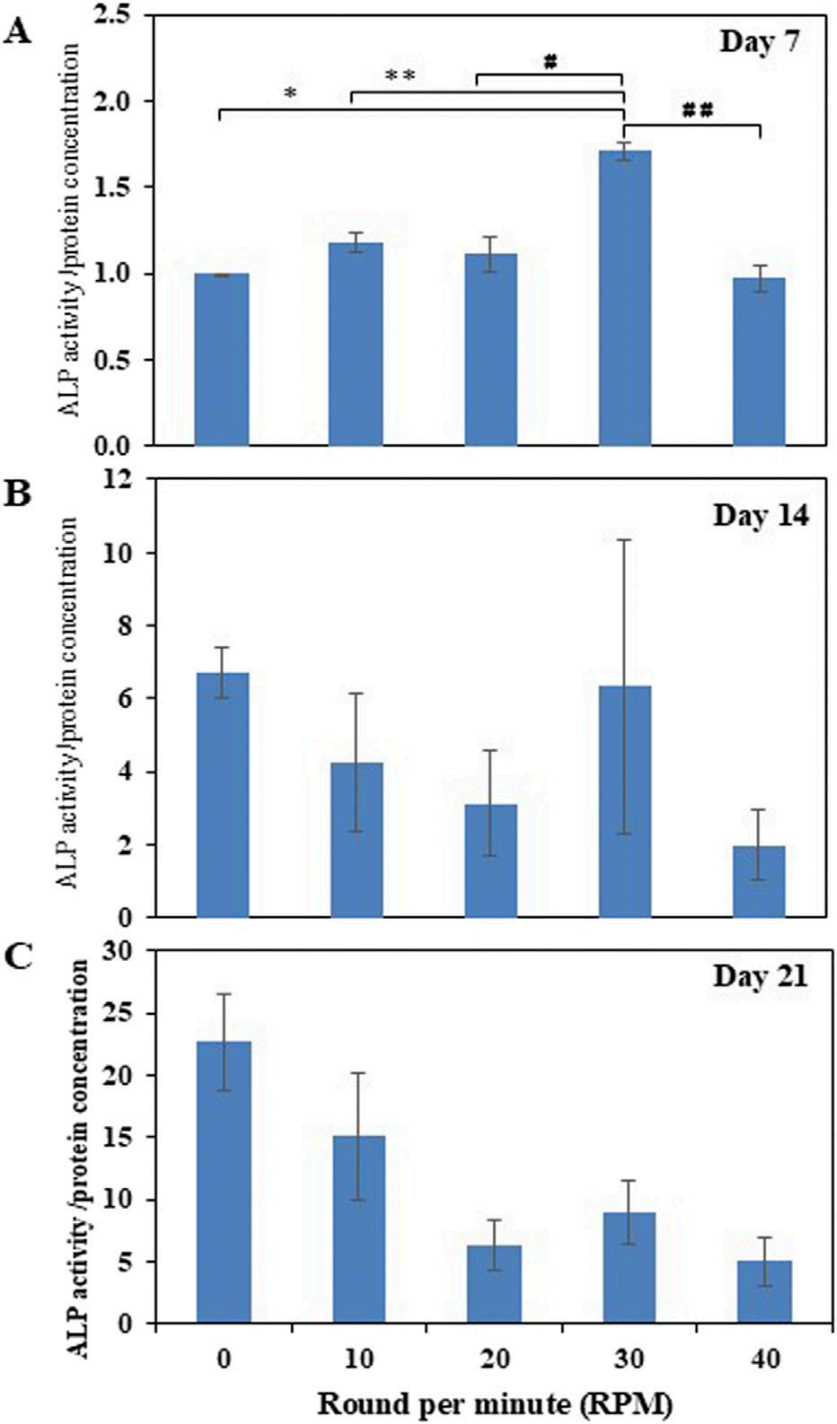
Normalized ALP activity/protein concentration (ng/mL) of the DPCs in the osteogenic induction medium was measured on days 7 **(A)**, 14 **(B)**, and 21 **(C)** of culture. * = *p* < 0.05 compared between the 0 RPM and 30 RPM groups, ** = *p* < 0.05 compared between the 10 RPM and 30 RPM groups, # = *p* < 0.05 compared between the 20 RPM and 30 RPM groups, ## = *p* < 0.05 compared between the 30 RPM and 40 RPM groups using an independent-samples Kruskal–Wallis test. Data are presented as mean ± SEM (*N* = 3, *n* = 3).

**FIGURE 6:**
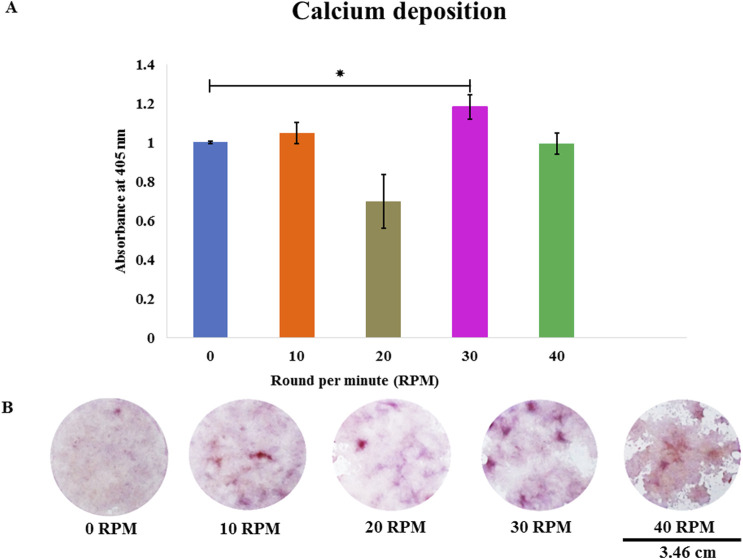
**(A)** Total calcium deposition of DPCs was assessed using alizarin red staining on day 21 of culture. **(B)** The photos show representative images of alizarin red staining at different OFF frequencies. Data are presented as mean ± SE, * = p < 0.05 compared between the 0 RPM group and 30 RPM group, using the independent-samples Kruskal–Wallis test. The scale bar represents 3.46 cm.

**FIGURE 7:**
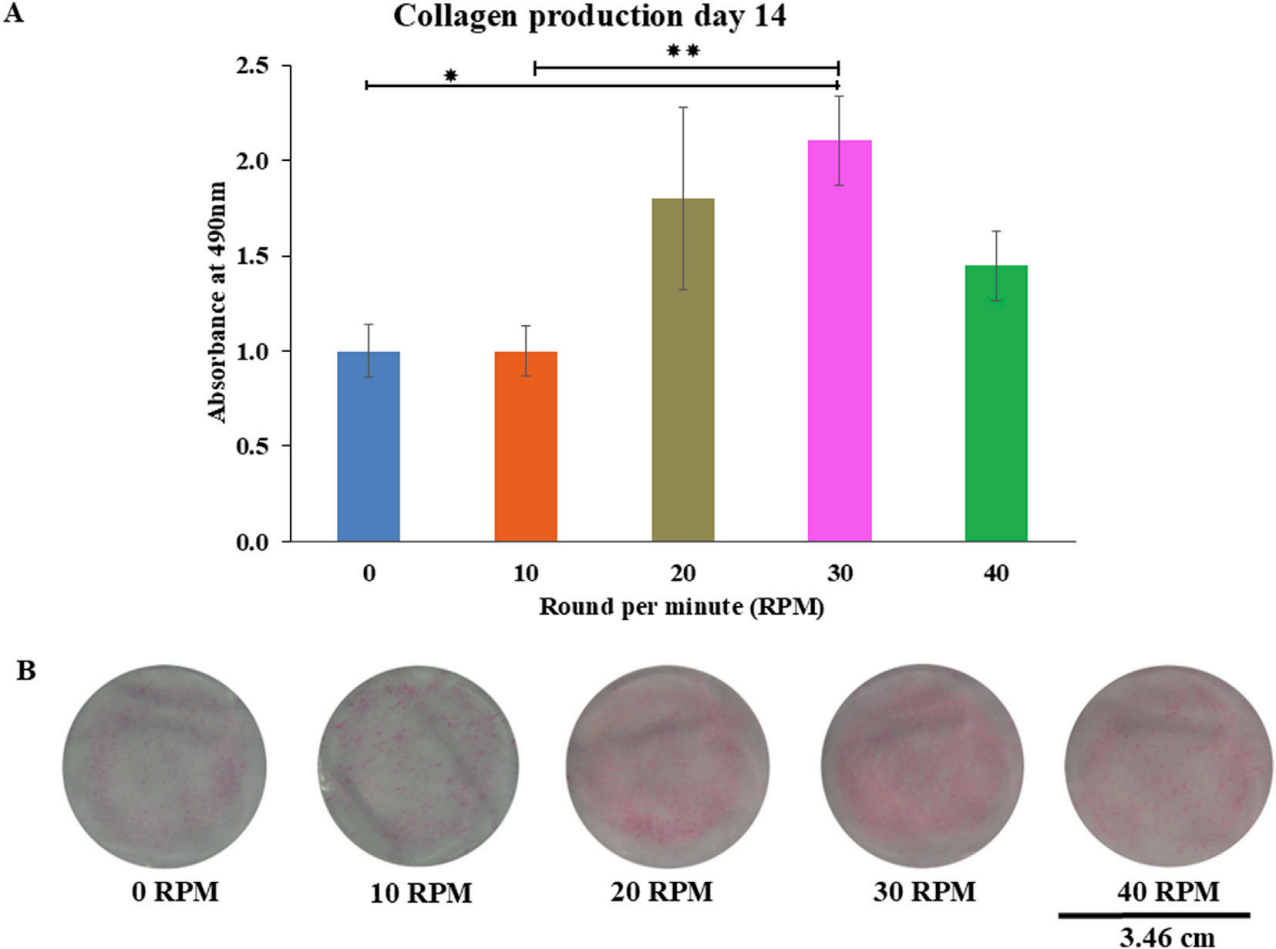
Collagen production of DPCs using picro-sirius red staining were measured **(A)** on 14 days of culture. The photo images showed representative sets of picro-sirius red staining varying by different oscillatory fluid flow (OFF) frequency **(B)**. Data were presented as mean ± SE, * = *p < 0.05* compared between the 0 RPM and 30 RPM groups, ** = *p < 0.05* compared between the 10 RPM and 30 RPM groups, using independent-samples Kruskal–Wallis test. The scale bar represents 3.46 cm.

**FIGURE 8:**
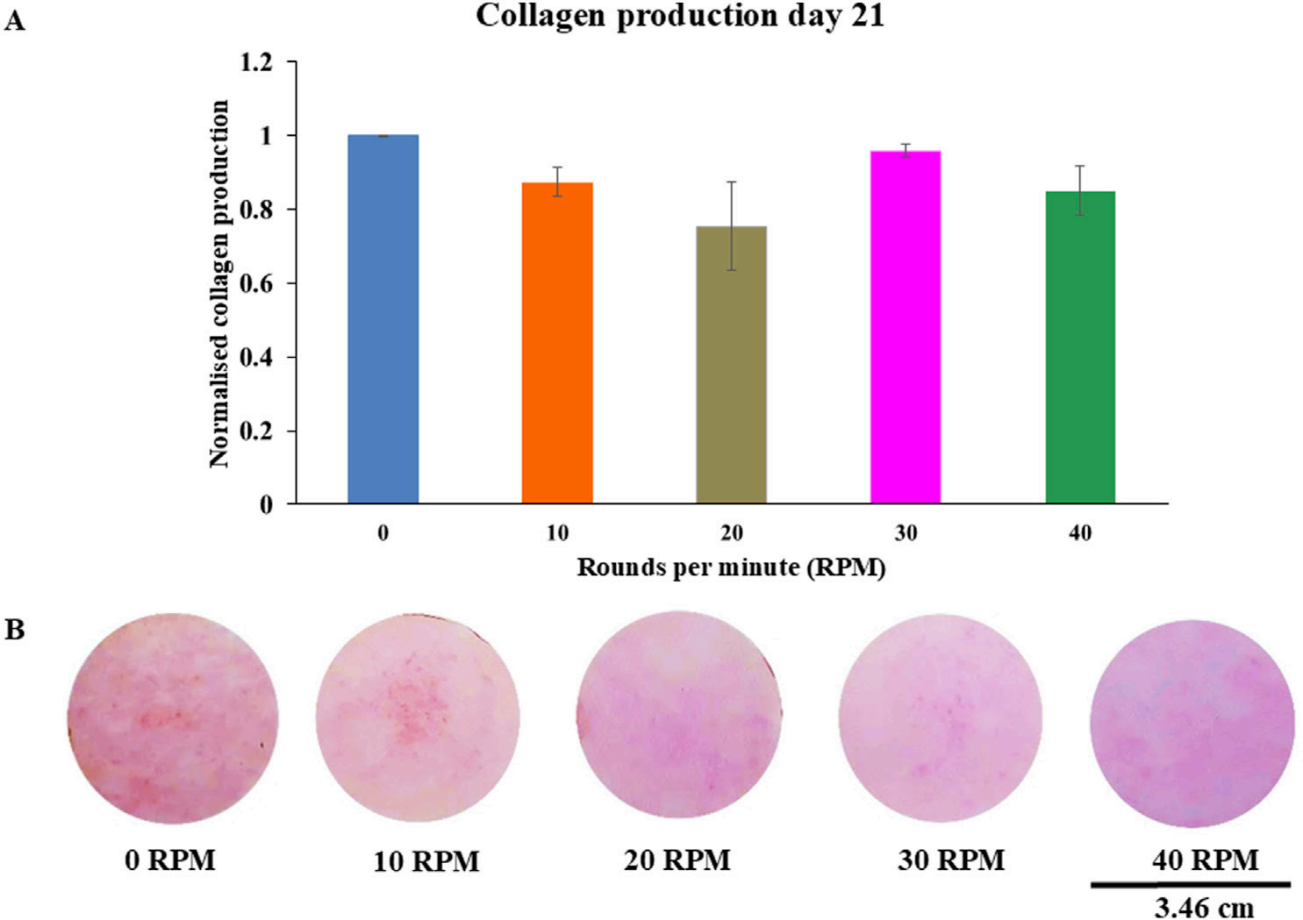
Collagen productions of DPCs using picro-sirius red staining were measured **(A)** on 21 days of culture. The photos showed representative sets of picro-sirius red staining varying by different OFF frequency **(B)**. Data were presented as mean ± SE, using independent-samples Kruskal–Wallis test. The scale bar represents 3.46 cm.

Total protein quantification of the DPCs was performed using the Pierce™ BCA Protein Assay Kit on Days 7, 14, and 21 of culture. Total protein increased continuously over the course of the 21 days in all experiments (Figure 4). The highest protein was found in the 30 RPM group, with no significant differences in total protein between the groups. The highest ALP activity on days 7 was observed in the 30 RPM group, with a significant difference between the groups *(p ≤ 0.05)* (Figure 5A). Total calcium mineralization of the DPCs was measured using alizarin red stain on Day 21 of culture. The highest calcium deposition was found in the 30 RPM group, which was significantly higher than the 0 RPM group *(p ≤ 0.05)* (Figures 6A, B). Total collagen production of the DPCs was measured using picro-sirius red stain on days 14 and 21 of culture. On day 14 of culture, the highest collagen production was found in the 30 RPM group, which was significantly higher than that the 0 RPM and 10 RPM groups *(p ≤ 0.05)* (Figures 7A, B). On Day 21 of culture, there were no significant differences between the groups (Figures 8A, B).

## 4 Discussion

This study showed that DPCs respond to OFF generated by an incubating rocking shaker as a mechanical stimulation by increasing ALP activity and calcium deposition in monolayer cultures. Bone tissue engineering may become an option for craniofacial reconstruction, and one crucial aspect is finding sources of cells for autologous bone formation.

This study evaluated various OFF frequencies in order to optimize osteogenesis of DPCs in monolayer culture. This study used human dental pulp tissue from 3 donors and was performed in triplicate. Jantapalaboon et al. (2018) successfully cultured DPCs under α-MEM media, and the DPCs demonstrated osteogenic differentiation on monolayer culture. Thus, this medium may facilitate DPCs in cell proliferation and differentiation into osteoblast-like cells. The current study is the first to investigate the response of DPCs to OFF using an incubating rocking shaker. A related study by the current author on human embryonic stem cell mesenchymal progenitors (hESMPs) and human jaw periosteal cells (HJPs) previously showed that OFF in an unheated rocking shaker could increase ALP activity, calcium mineralization, and collagen production (Puwanun et al., 2018).

Temperature is a vital environmental factor for cell cultures, and it affects the pH of cell culture media (Segeritz and Vallier, 2017). Thus the incubating rocking shaker was used in the current study to avoid temperature changes during the rocking periods.

DPCs in this study showed flow cytometry profiles that confirmed them to be MSCs. Consistent with numerous other studies, the expression of cell surface antigens CD90, CD105, CD146 was higher than 95%, indicating that the cell population contained MSCs. Additionally, CD45 expression was lower than 11% which would suggest the presence of heamatopoetic cells (Ha et al., 2021; Ma et al., 2021; Svandova et al., 2023).

It is well known that cells respond to mechanical stimuli through alterations in cell morphology and functionality. These alterations can depend on the magnitude of shear stress, the duration of cyclic load, and the frequency of loading (Mu et al., 2014). DPC morphology responded to mechanical stimulation in the 30 RPM group (1.24 Pa). Extrinsic mechanical factors, such as fluid flow, could regulate the osteogenic differentiation of MSCs (Steward and Kelly, 2015). They transduce mechanical forces into intracellular signaling (mechanotransduction), which varies with cell density and mediates cell differentiation. Mechanical signal perception in cells is mediated by mechanosensors, which influence cell-cell adhesion, ion channels, and cell-extracellular matrix (ECM) adhesion. Cell-cell adhesion plays a critical role in maintaining structural stability, allowing cells to be interconnected. The adhesion receptors responsible for cell-cell adhesion are cadherins.

A combination of Wnt/*β*-catenin, Mitogen-Activated Protein Kinase (MAPK), and Yes-associated protein (YAP)/transcriptional co-activator with PDZ-binding motif (TAZ) signaling pathways has demonstrated an important role in the differentiation of dental pulp stem cells (Zhao et al., 2023). The activation of Wnt/*β*-catenin signaling pathway in response to mechanical force can enhance cellular responses and induce cyclooxygenase (COX)-2 expression. COX has two isoforms: COX-1, and COX-2. COX-2 plays a crucial role in prostaglandin production to control bone cells’ response to mechanical loading. OFF might also stimulate bone cells’ endothelial nitric oxide synthase, which promotes bone formation. Cells’ endothelial nitric oxide synthase regulates nitric oxide (NO), which induces differentiation of osteoblasts and inhibits bone resorption by osteoclasts (Kraft et al., 2010).

Cell-ECM adhesions, including focal adhesions (FAs), have been demonstrated to physically connect cells with each other. Rens and Merks (2020) reported that cell-ECM interaction is an important factor in regulating the assembly and disassembly rate of FAs. A significant increase in the average number of FAs per cells was found in human dental pulp cells cultured in osteogenic induction media (Jones et al., 2015). YAP and TAZ activities increase depending on the amount of cell spreading and the stiffness of the substrate (Halbleib and Nelson, 2006). MAPK signaling pathway is regulated by the cytoskeletal. MARK can increase the expression of osteogenic markers such as osteocalcin, Bone morphogenetic protein 2 and ALP (Miyashita et al., 2017).

In a study by Raman et al. (2022) the number of FAs increased due to cell-ECM traction forces. As a result, cells expanded and elongated further as the stiffness of the substrate increased. The assembly of FAs resulted in the activation of intracellular calcium concentration and regulated mesenchymal stem cell differentiation. This is consistent with the cell morphology observed in the current study, which showed increased cell spreading and enhanced cell-cell adhesion. Similarly Sfriso et al. (2018), reported that cells cultured under fluid flow presented enhanced cell-cell communication by increasing the messenger molecules involved in synchronized interactions with neighboring cells. These factors enable DPCs to survive, proliferate, and differentiate (Marrelli et al., 2018).

Total protein quantification was found to be the same in all the experimental groups. These results suggest that OFF neither increased nor decreased cell proliferation in the monolayer cultured DPCs. The results of the current study are consistent with those of Liu et al. (2022) who subjected human exfoliated deciduous tooth pulp to centrifugal force using a microplate rotor with forces of 100, 200, and 300 g for 7 days and Nauman et al. (2001) who applied pulsatile fluid flow between 0.1 and 1.1 Pa from day 14 to day 21 while culturing rat femoral and tibial osteoprogenitor cells but found no effect on cell proliferation. Some studies showed the mechanical stimuli including a low-intensity pulsed ultrasound and low-density cyclic uniaxial compressive stress are able to increase the cell proliferation of human DPCs (Gao et al., 2017; Yang et al., 2018).

The ALP activity (the early markers for osteogenic differentiation) in this study increased over the course of the 21 days of culture. The 30 RPM group showed the most significant differences among the various groups on days 7 of culture. On day 21, the 30 RPM group showed decreased ALP activity compared to the other groups, while the 0 RPM group showed highest ALP activity. This is similar to the study by Kang et al. (2011), which showed an increase in ALP activity on day 7 after mechanical stimulation combined with ultrasonic. Delaine-Smith et al. (2012)’s study, which also showed the rocking group exhibited a high peak in ALP activity compared to the static group during the early stage of osteogenesis (day 14 of culture) and decreased in activity on day 21 (late stage of osteogenesis). ALP plays an important role in the initial stage of mineralization through its enzymatic hydrolysis activity which produces inorganic phosphate (Pi) from beta-glycerophosphate in cell culture media. Pi and calcium in OIM subsequently produce calcium phosphate deposition on the ECM. Therefore, ALP activity declines after the mineralization stage (Ansari et al., 2022).

A noteworthy result in the current study is that the 30 RPM group showed the highest calcium depositions (the late markers for osteogenic differentiation) of any group and this result was significantly higher than the control. A study by Huber et al. (2018) reported that low shear stress (<1 Pa), such as an extravascular flow or interstitial flow in the body, increased cell differentiation. James et al. (2015) reported that MSCs from bone marrow showed unique donor markers that control functions related to differentiation effectiveness. Some studies have also shown that even a low frequency of fluid flow (1 Hz and 1 h/day) can increase ALP and calcium deposition cells. For example, OFF of 2 Pa magnitudes and 2 Hz were found to induce osteogenic gene expression in murine MSC cell lines (Stavenschi et al., 2017). Nauman et al. (2001)’s study showed no effect on ALP activity or calcium deposition between the static group and various magnitudes of unidirectional pulsatile fluid flow (0, 0.06, and 0.6 Pa). The shear stress values of the OFF in this study were calculated using the method of Zhou et al. (2010). The shear stress values generated from 10, 20, 30, and 40 RPM were 0.50, 0.92, 1.24, and 1.34 Pa, respectively. In the current study, the 30 RPM group (1.24 Pa) had higher shear stress than reported in Nauman et al. (2001)’s study (0.6 Pa). Therefore, DPCs may respond to shear stress higher than 0.6 Pa by increasing osteogenesis.

Collagen production is one of the osteogenic markers of osteoprogenitor cells. On day 14 of culture, the 30 RPM group showed the highest collagen production of all groups, and this result was significantly higher than that of the 0 RPM and 10 RPM groups. On day 21 of culture, the results showed no significant differences in collagen production among the groups, which is consistent with the study by Zhou et al. (2011) used mechanical stimulation and was able to enhance a gene expression of collagen production at the middle stage of osteogenesis. This could explain why there was no difference in collagen production which this study was measured at day 21 (late stage for osteogenesis). In contrast, the study by Killinger et al. (2023), who found that collagen type I gene was not affected by uni-direction fluid flow using microfluidic plate flow. A study by Nohutcu et al. (1997) described how, under normal biological conditions, collagen type I gene would decrease collagen production before late mineralization. A report by Van Doren (2015) showed increased expression of matrix metalloproteinase-1 and matrix metalloproteinase-3 that degraded collagen through enzyme remodeling. This could explain why there was no difference in collagen production on day 21 of culture. However, an *in vitro* study by Han et al. (2008) reported that mechanical loading of DPCs using a cyclic strain stimulates increased production of both collagen and osteopontin. The physiological magnitude of fluid shear stress induced by pulsating fluid flow may mimic the normal bone situation *in vivo*. Loading osteocytes via the lacuno-canalicular network has been found to increase NO, prostaglandin E_2_ (PGE_2_), and cyclooxygenase (COX)-2 gene expression by bone cells *in vitro* (Kraft et al., 2010). Another study by Bakker et al. (2001) demonstrated that adult mouse long bone cells produced NO and PGE_2_ after stimulation with various shear stress from low, medium, and high fluid flow. The results showed that NO and PGE_2_ increased in a dose-dependent manner with increasing fluid flow shear stress (Bakker et al., 2001). They also compared the low fluid flow group using a higher viscous culture medium to the higher shear stress group. They found that NO and PGE_2_ levels increased to a level similar to the high fluid flow group using the normal viscosity culture medium. This means that NO and PGE_2_ depended on shear stress more than frequency.

Cell sheet tissue engineering is a technique used to support dense cell tissue in a sheet-like shape. This technique overcomes the limitation of traditional tissue engineering and allows for the delivery of more cells to the defect area. Recently, preclinical studies have reported on repairing organ damage, such as cleft palate and periodontitis defects in animal models (Hu et al., 2016; Hu et al., 2023). The cell sheet technique showed greater bone regeneration in the defect areas. Iwata et al. (2018) showed that autologous periodontal ligament-derived cell sheets combined with β-tricalcium phosphate bone fillers can improve clinical and radiographic outcomes in bone defects caused by periodontitis (gum disease), as demonstrated in a clinical study. This study found that applying OFF in an incubating rocking shaker at 30 RMP enhanced the mineralization of DPCs. Future research could involve inserting the mineralized cell sheet into *ex vivo*-cultured embryonic palatal shelves in an animal model to repair the defect, followed by clinical trials. For future treatment in regenerative dentistry, the cell sheet with higher mineralization could be placed on a denuded root, covered with a mucoperiosteal flap, and sutured tightly at the cemento-enamel junction (Hu et al., 2016).

## 5 Conclusion and suggestions

In this study, OFF did not affect cell proliferation, but it did increase ALP activity and mineralization. This finding is an important consideration, since the mineralization of DPCs could be enhanced by using an incubating rocking shaker at 30 RPM in the lab before implanting the DPCs into the patient for orofacial defect repair. In future work, it would be beneficial to have more donors of DPCs when measuring osteogenic potential to reduce donor variations. It might also be useful to run the current study again with a longer culture period (additional culture days) to see if this affects the results.

## Data Availability

The original contributions presented in the study are included in the article/Supplementary Material, further inquiries can be directed to the corresponding author.

## Supplementary material

SUPPLEMENTARY DATA S1 Phase contrast images show the cell morphology of human DPCs in OIM after 3, 7, and 14 days. On 3 days of culture, the cells in all groups were fibroblast-like and scattered individually. On 7 days of culture, In the 0 RPM (control) group, the cells were fibroblast-like and scattered individually. In the 10 RPM group and in the 20 RPM group, cells were scattered individually, similar to the control group. In the 30 RPM group, the cells were more clustered and gathered together. In the 40 RPM group, cells were polygonal in shape and more scattered in large spaces compared to the other groups. On 14 days of culture, the 20 RPM, 30 RPM, and 40 RPM groups were at confluence. The scale bar represents 200 μm.
