# Supplementary figures and images for: Oscillatory fluid flow enhanced mineralization of human dental pulp cells
Source: Front Bioeng Biotechnol. 2025 Jan 15;13:1500730. doi: 10.3389/fbioe.2025.1500730 (PMC11774892; doi:10.3389/fbioe.2025.1500730)

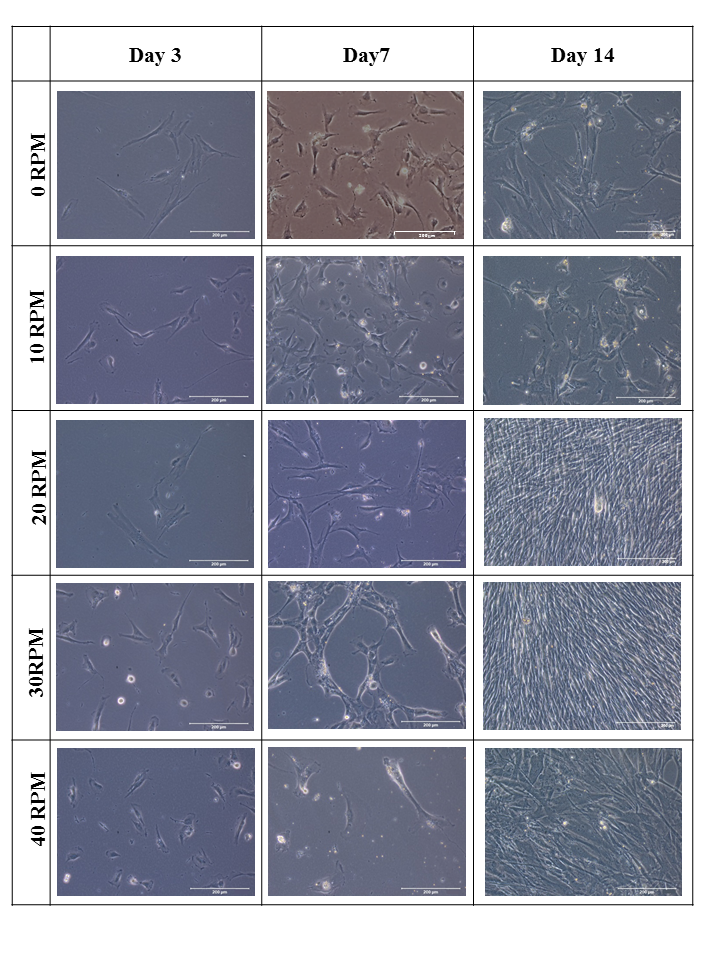

Supplement: Supplementary file 1 [file Image1.tif]
